# TcbZIP60 positively regulates pyrethrins biosynthesis in *Tanacetum cinerariifolium*

**DOI:** 10.3389/fpls.2023.1133912

**Published:** 2023-02-20

**Authors:** Zhizhuo Xu, Tuo Zeng, Jiawen Li, Li Zhou, Jinjin Li, Jing Luo, Riru Zheng, Yuanyuan Wang, Hao Hu, Caiyun Wang

**Affiliations:** ^1^ Key Laboratory for Biology of Horticultural Plants, Ministry of Education, College of Horticulture & Forestry Sciences, Huazhong Agricultural University, Wuhan, China; ^2^ School of Life Sciences, Guizhou Normal University, Guiyang, China

**Keywords:** *Tanacetum cinerariifolium*, pyrethrins biosynthesis, exogenous hormone, transcription factor, TcbZIP60

## Abstract

Pyrethrins, synthesized in the perennial plant *Tanacetum cinerariifolium*, are a class of terpene mixtures with high insecticidal activity and low human toxicity, which are widely used in plant-derived pesticides. Numerous studies have identified multiple pyrethrins biosynthesis enzymes, which can be enhanced by exogenous hormones such as methyl jasmonate (MeJA). However, the mechanism by which hormone signaling regulates pyrethrins biosynthesis and the potential involvement of certain transcription factors (TFs) remain unclear. In this study, we found that the expression level of a TF in *T. cinerariifolium* was significantly increased after treatment with plant hormones (MeJA, abscisic acid). Subsequent analysis identified this TF as a member of the basic region/leucine zipper (bZIP) family and was thus named *TcbZIP60*. TcbZIP60 was localized in the nucleus, suggesting that it is involved in the transcription process. The expression profiles of *TcbZIP60* were similar to those of pyrethrins synthesis genes in different flower organs and at different flowering stages. Furthermore, TcbZIP60 could directly bind to the E-box/G-box motifs in the promoters of the pyrethrins synthesis genes *TcCHS* and *TcAOC* to activate their expression. Transient overexpression of *TcbZIP60* increased the expression levels of pyrethrins biosynthesis genes, leading to the significant accumulation of pyrethrins. Silencing of *TcbZIP60* significantly downregulated pyrethrins accumulation and the expression of related genes. Overall, our results reveal a novel TF, TcbZIP60, that regulates both the terpenoid and jasmonic acid pathways of pyrethrins biosynthesis in *T. cinerariifolium*.

## 1 Introduction

*Tanacetum cinerariifolium* is an economically important horticultural ornamental plant in the Asteraceae family because it produces a class of insecticidal compounds called pyrethrins (Casida, 1973). Pyrethrins are widely used as plant-derived pesticides owing to their broad-spectrum and highly effective insecticidal activity, easy decomposition into harmless substances under light exposure, and low toxicity to mammals (Casida and Quistad, 1995). *T. cinerariifolium* has been grown as an environmentally friendly commercial crop in more than a dozen countries in Africa, Asia, Europe, and South America (Lybrand et al., 2020).

Natural pyrethrins are composed of six types of monoterpene esters with similar structures, including pyrethrin I and II, jasmolin I and II, and cinerin I and II (Ramirez et al., 2013). Pyrethrins are obtained by esterification between an acid moiety (pyrethric acid or chrysanthemic acid) and an alcohol moiety (pyrethrolone, cinerolone, or jasomolone) (Freemont et al., 2016; Khan et al., 2017). Monoterpene acids are derived from the methylerythritol-4-phosphate pathway. The first step of the reaction is the formation of chrysanthemyl diphosphate catalyzed by chrysanthemyl diphosphate synthase (CDS) from dimethylallyl diphosphate, which in turn is catalyzed by CDS to trans-chrysanthemol. CDS is a bifocal enzyme that is also known as chrysanthemol synthase (CHS) (Rivera et al., 2001; Yang et al., 2014; Hu et al., 2018; Lybrand et al., 2020). Subsequently, pyrethric acid is formed under the catalysis of alcohol dehydrogenase and aldehyde dehydrogenase (ALDH). Ketols are derived from the oxylipin alcohol pathway. In this way, linolenic acid is used as a substrate to form ketol through lipoxygenase, allene oxide synthase, allene oxide cyclase (AOC), oxo-phytodienoic acid reductase, jasmone hydroxylase, and pyrethrolone synthase (Song et al., 1993; Creelman and Mullet, 1997; Tijet and Brash, 2002; Ramirez et al., 2013; Li et al., 2018). Finally, pyrethrins are synthesized by the two precursor compounds under the catalysis of GDSL lipase-like protein (GLIP) (Kikuta et al., 2012). Although pyrethrins synthesis pathways have been largely clarified, there are relatively few studies on the mechanisms of pyrethrins synthesis at the transcriptional level.

Although pyrethrins are insecticidal compounds, on average, the pyrethrins content of the leaves of plants is only approximately 0.1% (dry weight), which is much lower than that of the flowers (1–2% dry weight) (Varga et al., 2021). A variety of methods have been evaluated to increase the yield of pyrethrins, including optimizing the cultivation mode or modifying the breeding strategy (Li et al., 2014). However, these strategies have not led to a major increase in the pyrethrins content of the leaves. Pyrethrins biosynthesis of the alcohol moiety derives from the jasmonic acid (JA) pathway (Hu et al., 2018). In particular, methyl jasmonate (MeJA) stimulates the overaccumulation of many secondary metabolites in plants. In a previous study, MeJA treatment was shown to induce the accumulation of pyrethrins for a short period, possibly *via* regulating multiple pyrethrins biosynthesis-related genes (Li et al., 2018). However, persistently treating MeJA to plants is not a robust strategy to maintain pyrethrins production.

In the process of plant evolution, many new transcription factor (TF) families have emerged during adaptation to changing environments or in response to exogenous hormones, further affecting the synthesis of secondary metabolites in plants by regulating the expression of target genes (Li et al., 2020; Chen et al., 2022). Therefore, the application of various hormones to plants is a feasible strategy to discover the critical TFs involved in pyrethrins biosynthesis. Basic leucine zipper (bZIP) is one of the most diverse TF families of plants, playing an important role in plant stress signal transduction, pathogen defense, and flower development (Wigge et al., 2005; Tang and Page, 2013; Sagor et al., 2015; Chen et al., 2021). All bZIP TFs possess a conserved bZIP domain, which is usually composed of 60–80 amino acids, including a basic DNA-binding region and a leucine (Leu) zipper domain that can recognize and combine cis-acting elements such as E-box (5′-CANNTG-3′), G-box (5′-CACGTG-3′), and ACGT-box (5′-ACGT-3′) in the gene promoters (Jakoby et al., 2002; Wolfgang et al., 2018).

Many studies have confirmed that bZIP TFs are involved in the response to signaling pathways and abiotic/biotic stress, including light signaling, abscisic acid (ABA) signaling, drought, and pathogen infections (Uno et al., 2000; Sornaraj et al., 2016; Wolfgang et al., 2018). Moreover, bZIP TFs also regulate the biosynthesis of numerous secondary metabolites in plants. For example, in *Artemisia annua*, the bZIP TF AaHY5 directly affects artemisinin biosynthesis through interaction with *AaCOP1* (Hao et al., 2019). In addition, AabZIP1 was reported to activate *ADS* and *CYP71AV1* gene expression under ABA treatment to promote artemisinin biosynthesis (Zhang et al., 2015). Along with a role in the biosynthesis of terpenoids, bZIP TFs also positively or negatively regulate flavonoids and alkaloids biosynthesis by activating the target genes in each biosynthesis pathway (Sibéril et al., 2001; Zhang et al., 2011; Pal et al., 2015; Mao et al., 2021). However, the downstream target genes of TFs are mainly involved in consecutive steps of metabolites synthesis or the same precursor-derived pathway. bZIP TFs regulating genes independently in different biosynthesis pathways are rarely reported.

In this study, we found that MeJA treatment activates reporter gene expression driven by pyrethrins biosynthesis-related gene promoters to increase the pyrethrins content of *T. cinerariifolium*. Based on these findings and the MeJA-treated transcriptome, we successfully identified a novel bZIP TF, named TcbZIP60. Further experiments demonstrated that TcbZIP60 responds to not only MeJA but also to a variety of other plant hormone signals, and can directly bind to the promoter region of the pyrethrins biosynthesis genes *TcCHS* and *TcAOC*. The transient overexpression and virus-induced gene silencing (VIGS) of *TcbZIP60* in *T. cinerariifolium* leaves further confirmed the positive role of TcbZIP60 in regulating the biosynthesis of both terpenoid- and JA-derived pyrethrins.

## 2 Materials and methods

### 2.1 Plant materials and growth conditions

*Tanacetum cinerariifolium* ‘W99’ plants grown in the flower base of Huazhong Agricultural University, Wuhan, China were used in this study. This cultivar has the advantages of easily rooting cuttings, rapid growth, and high pyrethrins content and, therefore, was suitable for the present experiments. W99 seedlings were subcultured in half-strength Murashige and Skoog medium for 1 month (25°C, 16 h light/8 h dark) prior to transient treatment with MeJA or ABA. The rooted cuttings were sprayed with 5 ml of 2 mM MeJA/ABA solution as a single foliar application. Leaves were sampled in triplicate at 0 (control), 2, 4, 6, 8, 12, and 24 h after treatment, immediately placed in liquid nitrogen, and stored at −80°C until further analysis.

Flower heads and leaves were harvested at seven flowering stages: S1, well-developed closed buds; S2, ray floret limb in vertical position; S3, ray floret limb in horizontal position and disc florets of the outermost whorl open; S4, three whorls of disc florets open; S5, all disc florets open; S6, termed the early overblown condition, the disc floret color is faded but the ray florets remain intact; S7, termed the late overblown condition, the disc florets retain little color and the ray florets are desiccated. Three biological replicates were collected for each sample, which were immediately frozen in liquid nitrogen and stored at −80°C for further analysis.

### 2.2 Exogenous MeJA effect on *TcCHS* and *TcGLIP* promoter activity

Our previously established transgenic chrysanthemum (*Chrysanthemum* × *morifolium*) harboring *TcCHS*-promoter-driven GFP and tobacco (*Nicotiana tabacum*) harboring *TcGLIP-*promoter-driven GUS were treated with MeJA (Sultana et al., 2015). The plants were sprayed with 5 ml of 300 μM MeJA dissolved in 0.8% ethanol as a single foliar application. Leaves were collected in triplicate at 0 (control) and 12 h after treatment, and were immediately frozen in liquid nitrogen. The GFP expression level was determined by quantitative real-time PCR (qRT-PCR) analysis. The GUS activity was measured as previously described (Luo et al., 2013).

### 2.3 Gene cloning and bioinformatic analysis

Total RNA was extracted from the collected samples using the standard phenol–chloroform extraction method. The first-strand cDNA was synthesized using the EasyScript^®^ One-step gDNA Removal and cDNA Synthesis SuperMix Kit (TransGen Biotech, Beijing, China), in accordance with the manufacturer’s instructions, using the total RNA extracts as the template. The *TcbZIP60* gene sequence of *T. cinerariifolium* was identified from the inflorescence transcriptome database determined by our laboratory. The Primer Premier software was used to design specific primers for amplifying the open reading frame (ORF) fragment. The primers are listed in **Supplementary Table S1**. The *cis*-acting elements of associated genes (*TcCHS*, *TcAOC*, *TcALDH*, and *TcGLIP*) were determined using the PlantCARE database. The *cis*-regulatory E-box and G-box elements were determined using PlantPAN 3.0 (Chow et al., 2019).

### 2.4 Real-time quantitative PCR

Total RNA was extracted using the Ultrapure RNA Kit (CWBIO, Beijing, China), and the EasyScript One-step gDNA Removal and cDNA Synthesis SuperMix Kit (TransGen Biotech) was used to reverse-transcribe the RNA into cDNA. A qRT-PCR analysis was performed using a LightCycler^®^ 96 Real-Time PCR System (Roche, Basel, Switzerland) in accordance with the manufacturer’s instructions, with SYBR Premix Ex Taq II (Takara, Kusatsu, Japan) and sequence-specific primers (listed in **Supplementary Table S1**). Glyceraldehyde-3-phosphate dehydrogenase (GADPH) was used as the internal reference gene (Li et al., 2019). The relative expression levels were calculated using the 2^−ΔΔ^ *^C^* ^t^ method with three biological and three technical replicates (Livak and Schmittgen, 2001). Student’s two-tailed *t-*test was used to determine statistical significance. Differences at *P* < 0.05 were considered to be significant and those at *P* < 0.01 were considered to be highly significant.

### 2.5 Phylogenetic analysis of TcbZIP60

The ORF of *TcbZIP60* was cloned from *T. cinerariifolium* cDNA using the sequence-specific primers TcbZIP60-F and TcbZIP60-R (**Supplementary Table S1**). *Arabidopsis* sequences homologous to the *TcbZIP60* sequence were identified by a Basic Local Alignment Search Tool (BLAST) search against The Arabidopsis Information Resource (TAIR) database, and homologous sequences from other plant species were identified by a BLAST search against the National Center for Biotechnology Information (NCBI) database. A phylogenetic tree was constructed with the maximum likelihood method using MEGA X software with 1000 bootstrap replications (Kumar et al., 2018). The GenBank accession numbers of the genes used in the analysis are listed in **Supplementary Tables S2 and S3**.

### 2.6 Subcellular localization of TcbZIP60

The *TcbZIP60* coding sequence was amplified by PCR using sequence-specific primers (**Supplementary Table S1**). The gene was cloned using the ClonExpress^®^ II One Step Cloning Kit (Vazyme Biotech, Nanjing, China). The full-length sequence of *TcbZIP60* (without the stop codon) was spliced into the pSuper-1300 GFP vector. The recombinant product was transferred into *Escherichia coli* strain DH5α, then sequenced, and the correct plasmid was extracted and constructed. The resultant plasmid was introduced into *Agrobacterium tumefaciens* strain GV3101, and then co-infiltrated into *Nicotiana benthamiana* leaves together with the RFP-NLS plasmid (a nuclear marker). The empty vector was used as a control. After 72 h of weak light exposure, fluorescence signals were observed with a confocal laser scanning microscope (TCS-SP8, Leica, Wetzlar, Germany).

### 2.7 Yeast one-hybrid assay

The full-length cDNA of *TcbZIP60* was cloned into the pGADT7 vector used for homologous recombination, and the promoter sequences of *TcCHS* and *TcAOC* were cloned separately into the pHis2.1 vector. The resultant pGADT7:*TcbZIP60* plasmid was co-transformed into yeast strain Y187, together with pHis2.1:*TcCHS* or pHis2.1:*TcAOC*, using the Super Yeast Transformation Kit (Coolaber, Beijing, China). The transformed yeast cells were selected on DDO (SD/−Leu/−Trp) medium and interreacted with TDO (SD/−Leu/−Trp/−His) medium at 30°C for 3 days.

### 2.8 Dual-luciferase reporter assay

To generate reporter constructs, and in accordance with the ORF sequence of the cloned *TcbZIP60* gene and the map of the plant expression vector, the downstream sequences of the gene promoter region (for *TcCHS* and *TcAOC*) were cloned into the linearized pGreenII0800-LUC vector. To generate effector constructs, the coding sequence of *TcbZIP60* was cloned into the linearized pGreenII62-SK vector under the control of the Cauliflower mosaic virus (CaMV) *35S* promoter. The resultant vectors were transiently co-expressed in *N. benthamiana* leaves. Luminescence was detected using the LB 985 Nightshade system (Berthold, Bad Wildbad, Germany). Introduction of the pGreen-62-SK empty vector and the pGreen-62-SK/pGreenII-0800-LUC : *TcCHS*/*TcAOC* constructs served as negative controls. Three biological replicates per treatment were measured.

The CDS of *TcbZIP60* was ligated into pGreenII62-SK vector to generate an effector plasmid, while *TcCHS*/*TcAOC* were fused into the vector pGreenII0800-LUC to produce the reporter plasmids. The effector, each of the two reporter constructs were co-transformed into *A. tumefaciens* GV3101, respectively. Transient expression assay in *N. benthamiana* leaves was as described previously (Geng and Liu, 2018). The activities of firefly luciferase (LUC) and Renilla luciferase (REN) were measured using the Dual-Luciferase^®^ Reporter Assay System (Promega, WI, USA). The promoter activity was expressed as the ratio of LUC to REN (**Supplementary Figure S1**).

### 2.9 Electrophoretic mobility shift assay

The ORF without the *TcbZIP60* terminator codon was used to generate the pET6×HN-C vector protein, which was fused into the N-terminal frame of 6×His, and the vector pET6×HN-C-TcbZIP60 was then transformed into *E. coli* strain Rosetta (DE3). For the induced recombinant protein, 0.5 mM IPTG was used, and the cultures were incubated at 18°C for 16 h. Then, Ni^2+^–nitrilotriacetic acid was used to purify the recombinant proteins. For the electrophoretic mobility shift assay (EMSA), the promoter fragments of *TcCHS* and *TcAOC* containing E-box or G-box *cis*-regulatory elements labeled with fluorescein amidite (FAM) as probes, the same but unlabeled DNA fragments, and *cis*-element mutant DNA fragments were used as competitors in the assay. After performing the EMSA assays, FAM-labeled DNA was detected from the chemiluminescent signal.

### 2.10 Transient overexpression of TcbZIP60 in *T. cinerariifolium* leaves

The full-length *TcbZIP60* coding sequence was cloned into the *Hin*dIII-linearized pGreenII62-SK vector downstream of the CaMV *35S* promoter using gene-specific primers (**Supplementary Table S1**). The experimental method followed a previously described procedure (Jung et al., 2015). The pGreenII62-SK : *TcbZIP60* vector was co-transformed together with the helper plasmid pSoup19 into *A. tumefaciens* strain GV3101. The pSoup19-transformed *Agrobacterium* cells were inoculated into YEB liquid medium containing 100 mg/l kanamycin and cultured on a rotating shaker at 28°C for 12 h. The supernatant was removed and resuspended in MES (containing 100 μM acetosyringone) to attain the final optical density (OD_600_ = 0.6). Leaves of *T. cinerariifolium* were placed in a 500 ml beaker containing a suspension of *A. tumefaciens*, and the beaker was then placed in a vacuum chamber at 0.23 ATM for 5 min. The soaked leaves were then dried with filter paper and stored in a petri dish lined with moist filter paper in the base. After 4 days of culture, leaves were sampled for qRT-PCR and high-performance liquid chromatography (HPLC) analyses. To determine the effect of transient expression, transient overexpression of GUS in *T. cinerariifolium* leaves was verified by staining with X-Gluc reagent (**Supplementary Figure S2**).

### 2.11 VIGS assay

The Tobacco rattle virus (TRV)-based vectors pTRV1 and pTRV2 were used for the VIGS assay (**Supplementary Table S1**). The pTRV2:*TcbZIP60* and pTRV1 plasmids were transformed into chemically active *A. tumefaciens* strain GV3101 cells using a liquid nitrogen freeze–thaw method. The positive transformant cells were inoculated into YEB liquid medium containing 100 mg/l kanamycin, incubated overnight on a shaker at 28°C, then reactivated in an infiltrating buffer (pH = 5.6) containing 10 mM MgCl_2_, 10 mM MES, and 100 µM acetoeugenone, and adjusted to OD_600_ = 0.6. After standing for 3 h, *Agrobacterium* cells containing the pTRV2 plasmid and pTRV1 plasmid were mixed (1:1, v/v), and the bacterial solution was injected through the adaxial epidermis of *T. cinerariifolium* leaves using a needle-free syringe. The leaves were then incubated in a culture room in the dark for 3 days and under light for 11 days. The pTRV1 and pTRV2 vectors were used as the control group. The method followed a previously described procedure (Senthil-Kumar and Mysore, 2014). The VIGS assay was conducted with three biological replicates. The empty vectors were used as controls. After 14 days, the samples were subjected to qRT-PCR and HPLC analyses.

### 2.12 Pyrethrins quantitation by HPLC

Transiently transformed leaf samples were dried for 48 h in an oven at 50°C to constant dry weight. The dried leaves were ground to a fine powder, then 100 mg powder was placed in a screw-capped glass tube, and dissolved in 600 μl *n*-hexane. After vortex-oscillation for 30 s, the sample was extracted in an ultrasonic water bath for a further 10 min, followed by vortex-oscillation for 30 s. The extracted samples were filtered with a 0.22 μm filter and analyzed by HPLC. The pyrethrins content was determined using a Waters HPLC system equipped with a photodiode array detector as described previously (Hu et al., 2018). Three biological replicates were analyzed for each sample, and commercial pyrethrum extract (Sigma-Aldrich, St. Louis, MO, USA) was used as the standard. Pyrethrins standard solution mother liquor was obtained by absorbing 3.0 μl pyrethrins standard and dissolving it in a small amount of *n*-hexane, and finally the volume was standardized to 1.00 ml. The mother liquor of 200, 100, 50, 25, 12.5, and 6.25 μl was standardized to 250 μl with methanol to obtain the pyrethrins standard sample solutions with concentrations of 1.15, 0.575, 0.288, 0.144, 0.072, and 0.036 mg/ml, respectively. After absorbing 20 μl of each standard sample solution, the samples were analyzed by HPLC and a standard curve for the pyrethrins standard samples was generated with Microsoft Excel.

### 2.13 Statistical analysis

All experiments were repeated using at least three biological replicates. The data were analyzed with one-way ANOVA and Student’s *t*-test using SPSS 18 software. Significant differences were determined with Student’s *t*-test, with *p* < 0.05 considered to be statistically significant.

## 3 Results

### 3.1 Exogenous MeJA positively regulates pyrethrins biosynthesis

**Figure 1:**
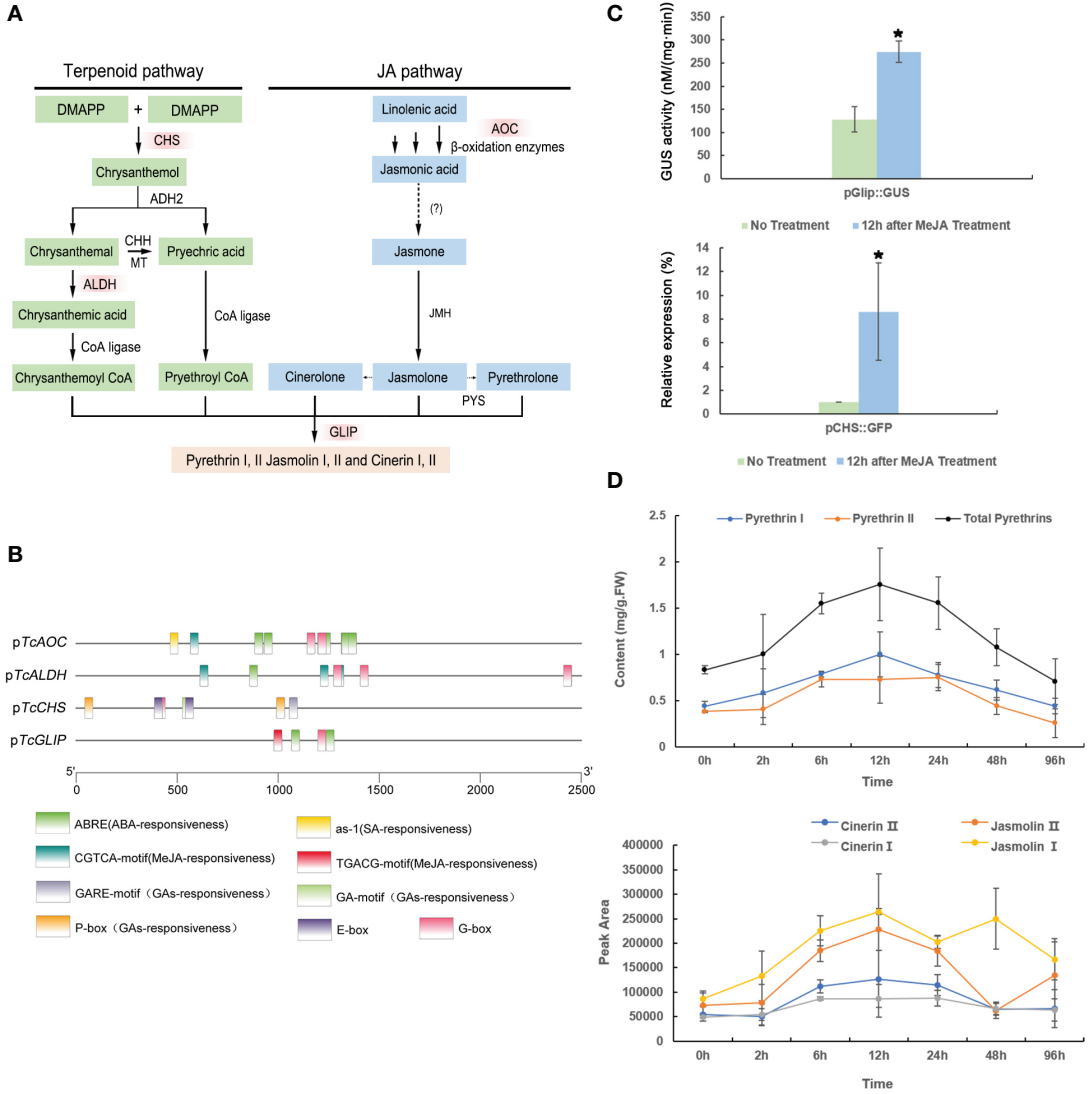
The pathway of pyrethrin biosynthesis and bioanalysis of promoters. **(A)** The biosynthesis of pyrethrins. The red boxes show the key genes involved in the pathway. **(B)** Analysis of the cis-regulatory elements in promoters of the key pyrethrin biosynthesis genes. **(C)** Effect of methyl jasmonate (MeJA) treatment on *CHS* and *GLIP* promoter expression. MeJA induced the pCHS::GFP gene in transgenic chrysanthemum. *GFP* gene expression was detected by real-time PCR at 12 h after spraying with 300 μM MeJA. MeJA induced the pGlip::GUS gene in transgenic tobacco. GUS activity was determined at 12 h after spraying 300 μM MeJA. Data are presented as mean ± SE. *P < 0.05 (ANOVA followed by Duncan’s multiple range test). **(D)** Effect of MeJA treatment on pyrethrins production in *Tanacetum cinerariifolium* plants. Leaves were collected at 2, 6, 12, 24, 48, and 96 h after spraying 300 μM MeJA. Samples at 0 h were considered the control. The pyrethrins content was quantified by high-performance liquid chromatography using pyrethrin external standards. Data indicate mean ± SE (n = 3).

Pyrethrins are derived from two independent pathways: the terpenoid biosynthesis pathway and the JA biosynthesis pathway (**Figure 1A**). Most pyrethrins biosynthesis enzymes have been identified to date, including those encoded by the key regulatory genes *CHS, AOC, GLIP*, and *ALDH* (Kikuta et al., 2012; Xu et al., 2018a). Therefore, we first analyzed the promoter elements of these key genes involved in pyrethrins synthesis using the PlantCARE and PlantPAN3.0 databases (**Figure 1B**), revealing several hormone-responsive elements, including those responding to MeJA, ABA, and salicylic acid (SA). This analysis indicated that MeJA treatment might play a role in pyrethrins synthesis.

Using our previously established transgenic platform for CHSpro-green fluorescent protein (GFP) and GLIPpro-b-glucuronidase (GUS) reporter genes (Sultana et al., 2015), we evaluated the MeJA-induced upregulation on the *CHS* and *GLIP* promoters individually. After treatment with 300 μM MeJA, the specific *GFP* expression level was measured by real-time polymerase chain reaction (PCR) and GUS activity was measured. Consequently, the *GFP* gene driven by the *CHS* promoter and the *GUS* gene driven by the *GLIP* promoter were both significantly activated at 12 h after MeJA treatment (**Figure 1C**), confirming that the *CHS* and *GLIP* promoters are MeJA-inducible, which will ultimately lead to enhanced pyrethrins production.

Indeed, in our previous study, we found that the pyrethrins content increased for a short time under high-concentration (2 mM) treatment of MeJA (Zeng et al., 2022). However, treatment of such a high concentration of MeJA leads to a dramatic decline in the pyrethrins content after 6 h due to disturbed homeostasis or metabolites feedback. Thus, in the present study, we treated *T. cinerariifolium* plants with a reduced concentration of MeJA of 300 μM. As expected, *T. cinerariifolium* plants showed a higher yield of pyrethrins after MeJA treatment, including all six constituents. The pyrethrins content increased by 2-fold to 0.99 mg/g per fresh weight at 12 h after MeJA treatment, and this accumulation level was maintained for 4 days (**Figure 1D**). These results strongly suggested that pyrethrins biosynthesis genes are MeJA-inducible and that the optimal working concentration of MeJA treatment could maintain a high pyrethrins yield for several days.

### 3.2 Cloning and characterization of bZIP60 in *T. cinerariifolium*

**Figure 2:**
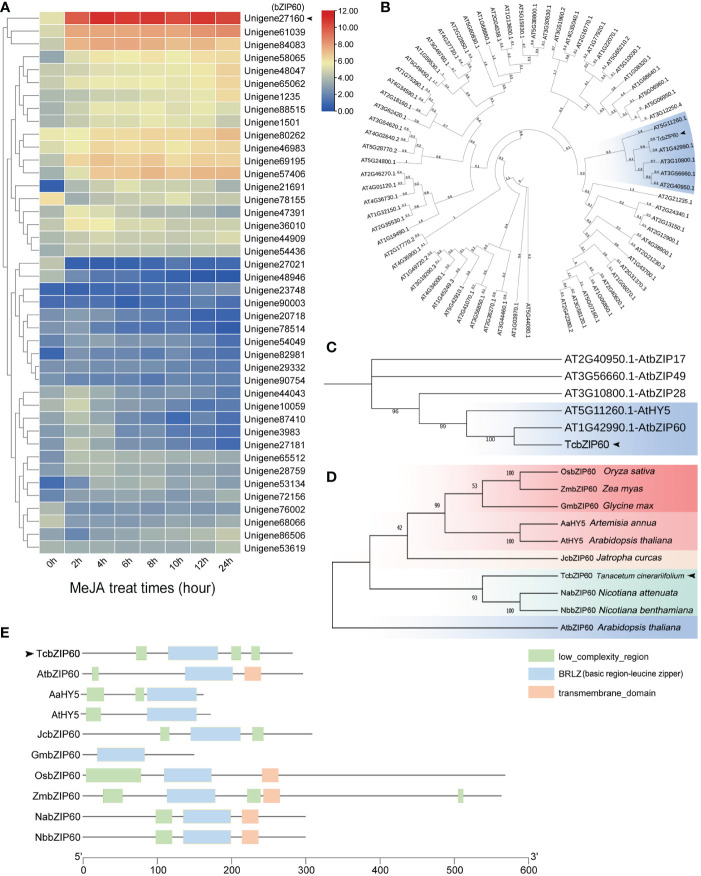
Expression patterns, phylogenetic analysis, and domain of the bZIP transcription factor family. **(A)** Heat maps of differentially expressed genes in the transcriptome with methyl jasmonate (MeJA) treatment to the *T. cinerariifolium* leaves at different times. The Pearson distance measure and Ward clustering algorithm were used. **(B)** Phylogenetic analysis of TcbZIP60 and *Arabidopsis thaliana* bZIP family protein sequences obtained from the PfamA domain database. **(C)** Partial enlargement of the phylogenetic tree in **(B)**. **(D)** Phylogenetic analysis of TcbZIP60 and bZIP family protein sequences of 12 other plants obtained from the NCBI database. **(E)** BRLZ domain of TcbZIP60 and its homologs in other species.

Considering that many E-box and G-box elements that bind to members of the bZIP TF family were identified in the promoter regions of pyrethrins biosynthesis genes, we searched for genes encoding putative bZIP TFs in the transcriptome data of *T. cinerariifolium*. Since we found that MeJA treatment can activate the *CHS* and *GLIP* promoter-driven reporter genes and improve pyrethrins production, we selected Unigene27160, which showed the highest expression level induced by MeJA, as the candidate bZIP TF (**Figure 2A**).

To identify the Unigene27160 gene in the *T. cinerariifolium* genome, the sequence was input as a query object in The Arabidopsis Information Resource (TAIR). In the phylogenetic comparison between Unigene27160 and members of the *Arabidopsis* bZIP TF family, Unigene27160 clustered with Arabidopsis *AtbZIP60* and *AtHY5* in the same evolutionary branch with high homology, suggesting that these genes exhibit similar functions (**Figure 2B**, blue box). Unigene27160 showed the closest relationship with *AtbZIP60* (**Figure 2C**). Moreover, 10 bZIP60 proteins from different plant species were selected for further comparative sequence analysis. These proteins clustered into five branches, represented by boxes with different colors in **Figure 2D**. Unigene27160 clustered with NabZIP60, NbbZIP60, and AtbZIP60, and contained a conserved bZIP domain similar to that of other bZIP proteins (**Figure 2E**). Thus, Unigene27160 was named *TcbZIP60*, and its open reading frame region was cloned for further analysis.

### 3.3 Expression profiles and subcellular localization of TcbZIP60

**Figure 3:**
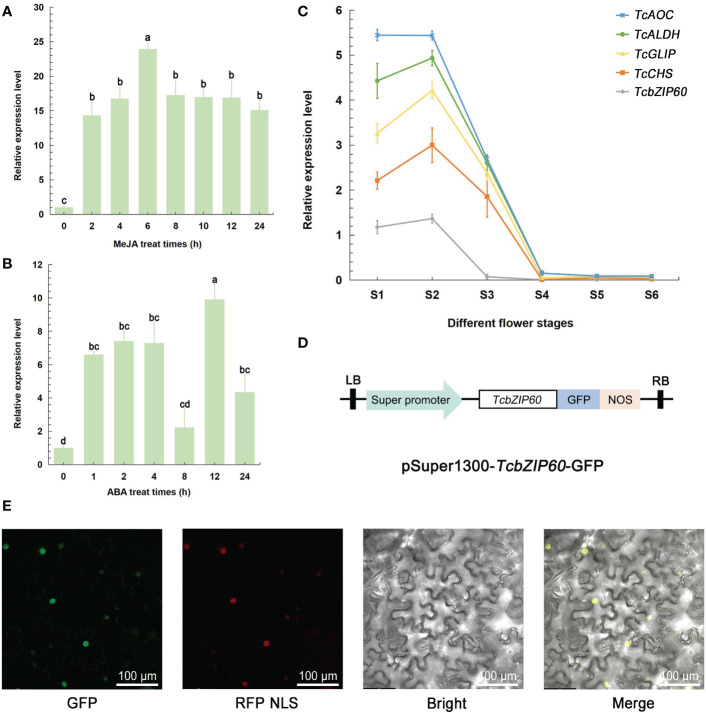
Expression profile and subcellular localization of TcbZIP60. **(A)** RT-qPCR analysis of the transcript abundance of *TcbZIP60* induced in the leaves of 1-month-old tissue culture seedlings treated with methyl jasmonate (MeJA); three biological replicates were established. **(B)** RT-qPCR analysis of the transcript abundance of *TcbZIP60* induced in the leaves of 1-month-old tissue culture seedlings treated with abscisic acid (ABA); three biological replicates were established. **(C)** RT-qPCR analysis of *TcbZIP60* transcript abundance from stages S1 to S6. **(D)** Diagram of the pSuper1300-*TcbZIP60*-GFP construct. **(E)** Subcellular localization of TcbZIP60 in *N. benthamiana* leaves. Scale bars = 100 μm.

Since *TcbZIP60* expression was most strongly induced by MeJA, we confirmed this result by real-time PCR and further investigated whether other hormones could also induce the expression of *TcbZIP60*. As expected, treatment with both MeJA and ABA strongly induced *TcbZIP60* expression, and high-level expression was maintained for 24 h (**Figures 3A, B**). To confirm that TcbZIP60 is involved in pyrethrins biosynthesis, the relative expression levels of *TcbZIP60* and other pyrethrins biosynthesis genes were analyzed at different flowering stages. The expression profiles were similar, with high expression detected in the first two stages (**Figure 3C**). Based on these results, we speculated that the TF TcbZIP60 regulates pyrethrins synthesis through interaction with the pyrethrins synthase gene.

To determine the subcellular localization of TcbZIP60, the coding sequence of *TcbZIP60* was fused into the GFP framework under control of the *CaMV35S* promoter. When TcbZIP60-GFP was instantaneously expressed in *Nicotiana benthamiana* leaves, strong and specific fluorescence was observed in the nucleus (**Figure 3D**). In addition, the co-localization of the GFP signal and red fluorescent protein (as a nuclear localization signal) resulted in a fused yellow fluorescence signal (**Figure 3E**). These results suggested that TcbZIP60 is a nuclear-localized protein, further supporting its potential role as a TF.

### 3.4 TcbZIP60 directly binds to the *TcCHS* and *TcAOC* promoters

**Figure 4:**
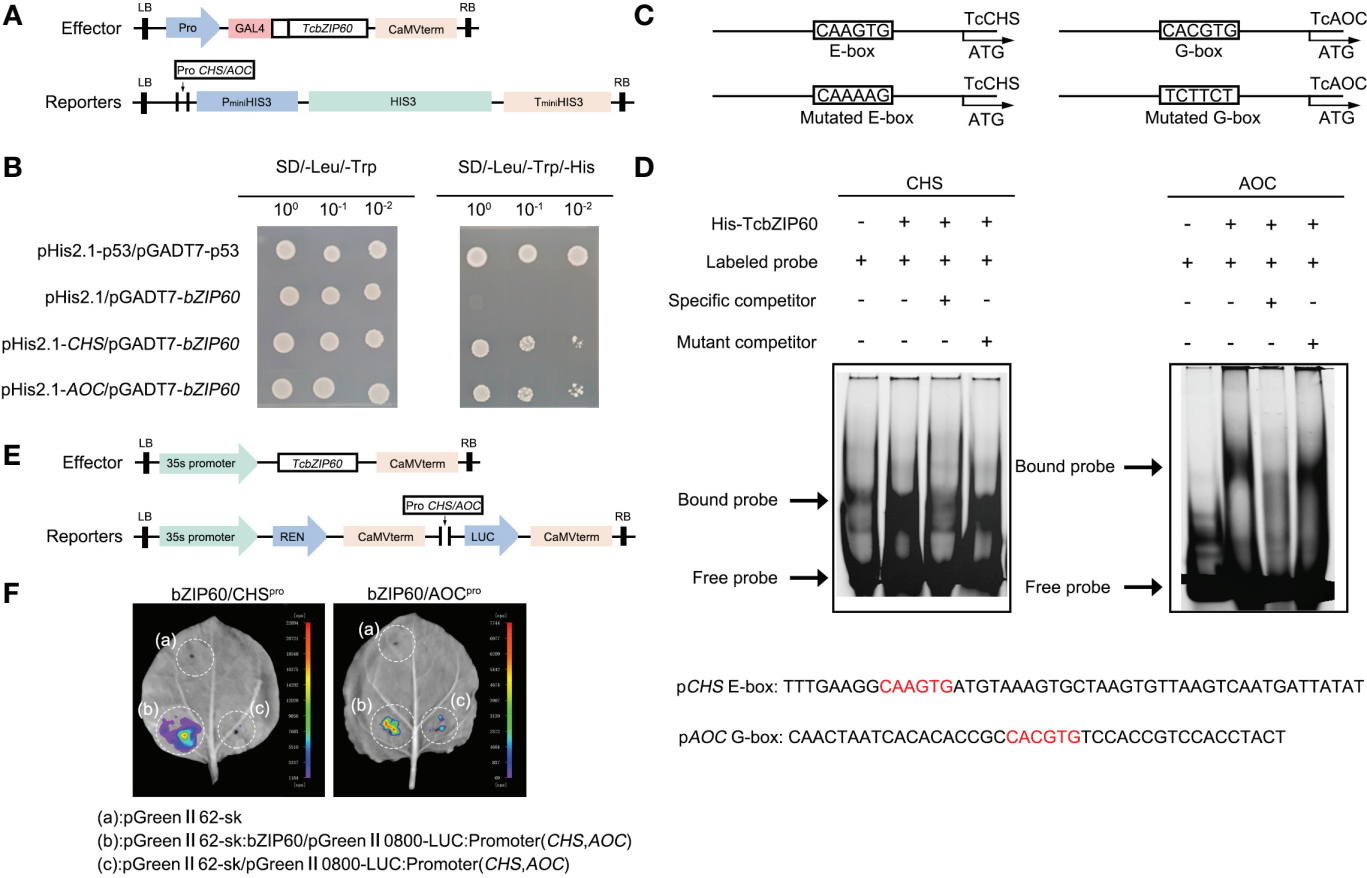
TcbZIP60 directly binds to and activates the promoters of the *TcCHS* and *TcAOC* genes. **(A)** Diagram of vectors used in the yeast one hybrid (Y1H) assay. Reporters: the promoter of *TcCHS*/*TcAOC* was introduced into the pHis2.1 vector. Effector: *TcbZIP60* was introduced into the pGADT7 vector. **(B)** Y1H assay showing TcbZIP60 binding to *TcCHS*/*TcAOC* promoter fragments. **(C)** Schematic diagram of the *TcCHS*/*TcAOC* promoter showing the potential TcbZIP60 binding sites. The predicted E-box/G-box and mutated E-box/G-box sites and sequences are indicated with black boxes. **(D)** Electrophoretic mobility shift assay (EMSA) of interactions between TcbZIP60 and the promoters of the *TcCHS* and *TcAOC* genes. The FAM-labeled probe (10 μM) was competed with a 50-fold excess of unlabeled wild-type cold probes and unlabeled mutant-type cold probes. **(E)** Diagram of dual-LUC vectors. Effector: *TcbZIP60* was introduced into the pGreen-62-SK vector, which was driven by the 35S promoter. Reporters: the promoter of *TcCHS*/*TcAOC* was introduced into the pGreen II-0800-LUC vector, driving the translation of LUC. **(F)** Dual-LUC reporter assay showing TcbZIP60 binding to the *TcCHS*/*TcAOC* promoters and promotion of LUC expression.

The binding sites of bZIP TFs were predicted in the promoter sequence of the pyrethrins synthesis genes (**Figure 1B**). In *T. cinerariifolium*, similar expression profiles were observed between *TcbZIP60* and pyrethrins synthesis genes during different flowering stages (**Figure 3C**). Therefore, we further speculated that TcbZIP60 can regulate the expression of these genes by directly binding to the corresponding promoters. To test this hypothesis, we performed a yeast monohybrid (Y1H) experiment. The promoters of pyrethrins synthesis genes were cloned and inserted into the pHis2.1 vector to generate reporter genes. TcbZIP60 was fused to the GAL4 activation domain to generate the effector construct pGADT7-*TcbZIP60* (**Figure 4A**).

In the interaction experiments with several pyrethrins synthesis gene promoters, yeast clones were observed on solid synthesized Leu, Trp, and His (SD/-Leu-Trp-His) media only when pGADT7-*TcbZIP60* was transferred into yeast cells expressing pHis2.1-*TcCHS* and pHis2.1-*TcAOC*, but not in those expressing pHis2.1. This suggested that TcbZIP60 binds directly to the promoters of *TcCHS* and *TcAOC* (**Figure 4B**). In addition, we hypothesized that TcbZIP60 directly binds to the motifs on the promoters of the two genes, which was further verified by electrophoretic mobility shift assays (EMSAs).

We predicted that TcbZIP60 binds to the potential E-box and G-box sites on the two promoters, and designed the corresponding mutation sites (**Figure 4C**). The migration bands were detected in the presence of the purified and concentrated TcbZIP60 protein and the labeled probe of the E-box containing the *TcCHS* promoter or the G-box containing the *TcAOC* promoter (**Figure 4D**). When 50 times the concentration of the cold probe (unlabeled probe) was added, the intensity of the migration band decreased. When the mutant probe (unlabeled probe) was added, the intensity of the migration band recovered.

To further verify the interaction between TcbZIP60 and the *TcCHS* or *TcAOC* promoter in plants, we performed a transient dual-luciferase (dual-LUC) assay with a reporter structure (*TcCHS*/*TcAOC* Pro : LUC) and effector structure (**Figure 4E**). Strong LUC activity was observed in tobacco leaves co-transformed by *TcCHS*/*TcAOC* Pro : LUC and 35S:*TcbZIP60*. However, almost no fluorescence signal was detected in tobacco leaves co-transformed with *TcCHS*/*TcAOC* Pro : LUC and an empty carrier (**Figure 4F**). These results suggested that the TF TcbZIP60 directly activates promoters of the *TcCHS* and *TcAOC* genes and regulates pyrethrins biosynthesis in *T. cinerariifolium*.

### 3.5 Activation of pyrethrins biosynthesis by TcbZIP60

**Figure 5:**
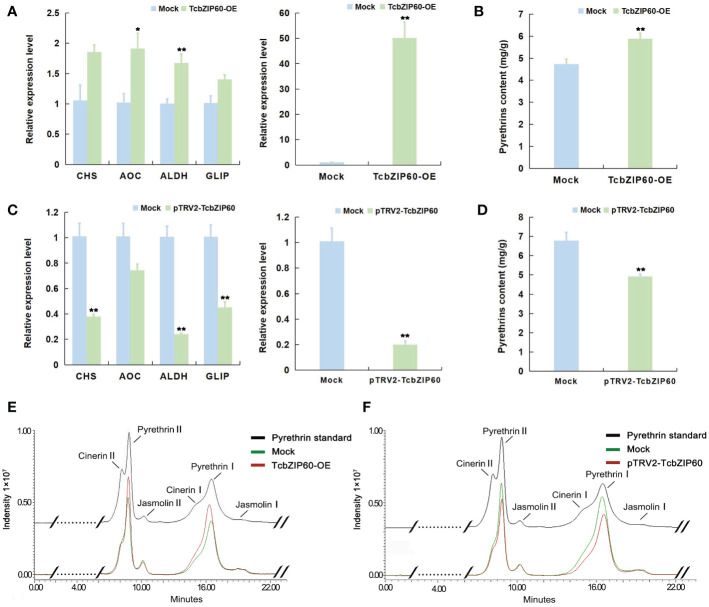
Transient overexpression and TRV-VIGS treatment of *TcbZIP60* in *T. cinerariifolium* leaves. **(A)** Relative expression levels of pyrethrin biosynthesis genes in *T. cinerariifolium* leaves at 4 days after transient overexpression of *TcbZIP60*. Mock: transient overexpression of the empty vector; *TcbZIP60*-OE: transient overexpression of pGreen-62-SK-*TcbZIP60*. The value for the Mock was set to 1. **(B)** Quantity of pyrethrins in *T. cinerariifolium* leaves after transient *TcbZIP60* overexpression at 4 days. The ordinate represents the pyrethrins content measured by high-performance liquid chromatography (HPLC) and the abscissa represents the six major constituent pyrethrins. **(C)** Relative expression levels of pyrethrins biosynthesis genes in *T. cinerariifolium* leaves at 4 days after TRV-VIGS treatment of *TcbZIP60* at 14 days. Mock: VIGS empty pTRV2 vector; pTRV2-*TcbZIP60*: *TcbZIP60* silencing of leaves. The value for Mock was set to 1. **(D)** Quantity of pyrethrins in *T. cinerariifolium* leaves after TRV-VIGS treatment of *TcbZIP60*. The ordinate represents pyrethrins content measured by HPLC and the abscissa represents the six major constituent pyrethrins. **(E)** Representative chromatogram of total pyrethrins showing the peaks of six different constituents (cinerarin II, pyrethrin II, jasmoline II, cinerarin I, pyrethrin I, and jasmoline I) in a standard sample (black line), *TcbZIP60*-OE (red line), and Mock (green line). **(F)** Representative chromatogram of total pyrethrins showing the peaks of six different constituents in a standard sample (black line), Mock (green line), and pTRV2-*TcbZIP60* (red line). Asterisks indicate that the value is significantly different from that of the control (**P<0.01, *P < 0.05).

Previous studies demonstrated that bZIP TFs in *A. annua* bind to gene promoters and regulate artemisinin biosynthesis (Hao et al., 2019; Lv et al., 2019). Similarly, we confirmed that TcbZIP60 can directly bind to the promoters of the pyrethrins biosynthesis genes *TcCHS* and *TcAOC*. Therefore, to further verify the role of TcbZIP60 in regulating pyrethrins biosynthesis, we constructed *TcbZIP60*-overexpressing plants (*TcbZIP60*-OE) and *TcbZI60*-suppressed plants (pTRV2-*TcbZIP60*) in *T. cinerariifolium*. Transient overexpression and transient RNA interference-mediated silencing of TcbZIP60 were performed in the leaves of *T. cinerariifolium*, and the expression of the pyrethrins biosynthesis genes was detected by reverse transcription-quantitative PCR after transformation. Compared with wild-type (Mock) plants of pGreenII 62-SK, the transcription level of *TcbZIP60* in *TcbZIP60*-OE plants significantly increased by 49.65 times, and the expression levels of the pyrethrins synthesis genes *TcCHS* and *TcAOC* also increased by 1.76 times and 1.88 times, respectively. The other two pyrethrins biosynthesis genes, *TcALDH* and *TcGLIP*, were also upregulated with *TcbZIP60* overexpression (**Figure 5A**). By contrast, in the *TcbZIP60*-silenced plants, the transcription level of *TcbZIP60* decreased by 0.8-fold (**Figure 5B**) and the expression levels of four pyrethrins synthesis genes also decreased (**Figure 5C**).

Subsequently, we measured the contents of pyrethrins in the transient overexpression and RNA interference plants. We used the pyrethrins standard to compare and identify the six main components of pyrethrins in the sample as shown by the arrow and the black line in **Figures 5E, F**, whereas the red and green lines represent the HPLC results of the experimental group and the control group, respectively. Compared with the pGreenII 62-SK Mock plants in the blank control, the total amount of pyrethrins in the overexpression plants significantly increased by 1.239 times (**Figures 5B, E**) and decreased by 0.273 times in the *TcbZIP60* interference plants (**Figures 5D, F**). These results showed that TcZIP60 can positively regulate pyrethrins accumulation by activating pyrethrins biosynthesis genes.

## 4 Discussion

Pyrethrins represent unique metabolites produced in pyrethrums that can provide plants with an effective endogenous chemical defense against insect and fungal diseases (Zeng et al., 2021). Since the 19th century, pyrethrins products have been gradually used as household and agricultural insecticides, and as insect-borne disease prevention agents (Orenstein, 1913; Lange and Akesson, 1973; Katsuda, 1999). However, in the extraction process for agricultural production, pyrethrins are obtained from the dry flowers of *T. cinerariifolium* and the yield of pyrethrins per plant is low, only accounting for 0.10–1.35% of the dry weight of flowers (Varga et al., 2021). Therefore, increasing the amount of pyrethrins produced in the leaves is the key to breaking through the bottleneck of production.

Previously, we reported that MeJA can upregulate the expression of pyrethrins biosynthesis genes as well as induce pyrethrins accumulation (Zeng et al., 2022). However, the high working concentration of MeJA used in the previous study cannot be sustained to constituently increase the pyrethrins content. In general, under stress/MeJA treatment, plant growth and metabolites synthesis will be halted to invest in defense. In the present study, treatment of a lower concentration of MeJA maintained the increase in the pyrethrins content in the leaves for more than 3 days. This was considered to be due to activation of the promoters of pyrethrins biosynthesis genes by MeJA. To further investigate the mechanism by which MeJA regulates pyrethrins biosynthesis, we identified the TF TcbZIP60 by the global expression profile, which was confirmed to be induced by exogenous MeJA, suggesting that TcbZIP60 might be involved in MeJA-regulated pyrethrins biosynthesis.

In addition to MeJA, ABA also plays an important role in regulating the biosynthesis of terpenoids (Jing et al., 2009). TFs involved in mediating ABA stimulation of terpenoids biosynthesis, including AaHY5, AaTGA6, and AabZIP1, respond to JA, SA, and ABA treatment and regulate artemisinin biosynthesis in *A. annua* (Hao et al., 2019; Lv et al., 2019; Shu et al., 2022). Similarly, we found that the transcription level of *TcbZIP60* was regulated by ABA treatment. Cross-talk between ABA and MeJA is a universal phenomenon, and some TFs involved in ABA signaling also participate in MeJA signaling in Arabidopsis and tobacco (Lackman et al., 2011). For example, AaMYC2, which is involved in JA signaling, was suggested to function as the bridge of JA signaling to the ABA signaling pathway (Abe et al., 2003). *AaMYC2* expression could be upregulated by ABA-induced AabZIP1 through direct interaction to subsequently activate *AaALDH1* transcription, thereby governing artemisinin biosynthesis (Shu et al., 2022). In *T. cinerariifolium*, TcMYC2 was also identified as a MeJA-induced TF that positively regulates pyrethrins biosynthesis by upregulating *TcCHS*, *TcAOC*, and *TcGLIP* gene expression (Zeng et al., 2022). Thus, it is strongly speculated that TcbZIP60 might also interact with TcMYC2 to co-regulate pyrethrins biosynthesis. This could explain why we detected upregulation of *TcGLIP* expression even when its promoter was not directly activated by TcbZIP60. Therefore, whether more TFs in *T. cinerariifolium* function as a network to cooperatively regulate pyrethrins biosynthesis is worthy of further study.

Through the development of metabolic engineering, there has been significant progress in producing pyrethrins in other model plants by introducing pyrethrins biosynthesis genes into heterologous systems. However, to date, only the precursors of pyrethrins have been synthesized in tobacco and tomato (Xu et al., 2018b; Xu et al., 2019), because not all of the genes in the full pathway have been cloned. *T. cinerariifolium* remains the only available resource for pyrethrins production. Because pyrethrins biosynthesis is derived from two independent pathways involving multiple enzymes, overexpression of one or two enzymes would not be efficient in increasing pyrethrins biosynthesis, and it is even harder to co-express enzymes from different pathways simultaneously. Members of the bZIP TF family typically target the key steps in the biosynthesis of secondary metabolites. bZIP TFs contain a conserved alkaline region consisting of 16 amino acid residues with a constant N-X7-R/K motif, which can recognize and bind to the specific elements on the DNA sequence on the promoters, thereby affecting the multiple genes involved in the synthesis of secondary metabolites. Thus, overexpression of bZIP TFs leads to the improvement of secondary metabolites production. For example, in *A. annua*, AabZIP1 binds to both the *AaADS* and *AaCYP71AV1* promoters, and increases the expression of *AaADS* and *AaCYP71AV1* as well as the artemisinin content (Zhang et al., 2015). Overexpression of *AaHY5* and other upstream transcriptional regulatory genes significantly increased the transcriptional levels of the downstream genes *AaADS*, *AaCYP71AV1*, *AaDBR2*, and *AaALDH1*, and increased the synthesis and accumulation of secondary metabolites (Hao et al., 2019). In *Oryza sativa*, bZIP72 binds to the *AOC* promoter G-box, which strengthens the *AOC* transcription level and endogenous JA level (Wang et al., 2020). In *T. cinerariifolium*, *CHS* and *AOC* are upstream genes that are more likely to become rate-limiting factors for pyrethrins synthesis (**Figure 1A**). AOC is considered to be the first key enzyme in JA synthesis, affecting the overall biosynthesis rate (Yoeun et al., 2018), and CHS is the first key enzyme in the biosynthesis of pyrethrins (Hu et al., 2018). Our results demonstrated that TcbZIP60 could bind to the E-box and G-box cis-elements on the promoters of both *TcCHS* and *TcAOC*, and activated the transcription of these genes *in vitro*. Moreover, to further confirm the function of TcbZIP60 in pyrethrins production, we transiently overexpressed the *TcbZIP60* gene in *T. cinerariifolium* leaves, which upregulated the expression of the main pyrethrins biosynthesis genes *TcCHS*, *TcAOC*, *TcALDH*, and *TcGLIP*, consequently leading to the significant accumulation of pyrethrins. As expected, interference of *TcbZIP60* expression in *T. cinerariifolium* decreased the production of pyrethrins.

**Figure 6:**
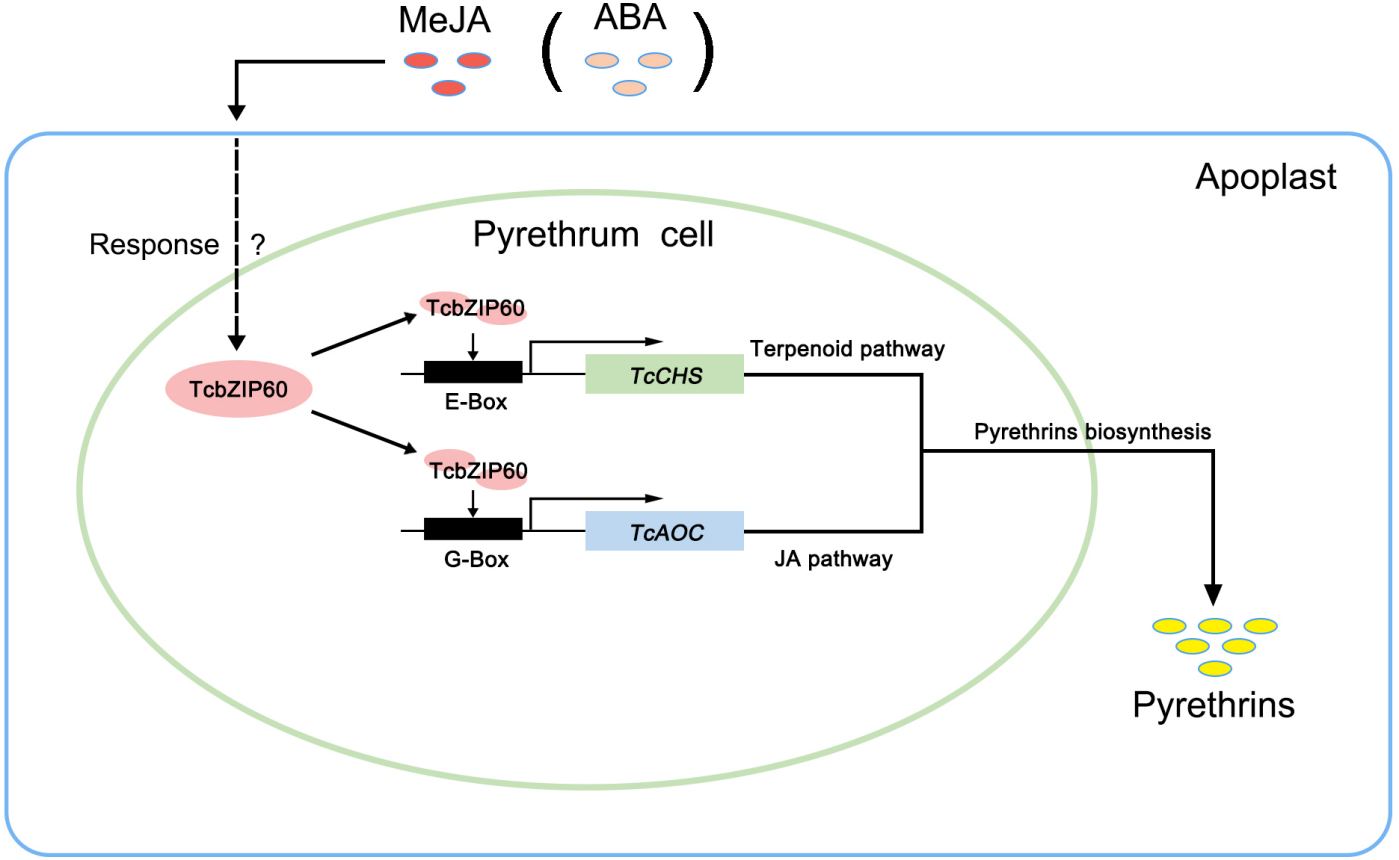
Model illustrating the involvement of TcbZIP60 in regulating pyrethrins biosynthesis in *T. cinerariifolium*. The arrows indicate TcbZIP60, regulated by methyl jasmonate (MeJA) or abscisic acid (ABA) signals, activating the *TcCHS* and *TcAOC* genes by binding to their promoters and subsequently regulating pyrethrins biosynthesis. JA, jasmonic acid.

Based on these results, we propose a model of the role of TcbZIP60 in the regulation of pyrethrins biosynthesis in *T. cinerariifolium* (**Figure 6**). This study thus identified TcbZIP60 as a novel and important positive regulator that could be used in engineering pyrethrins synthesis to improve its production in *T. cinerariifolium*.

## Data availability statement

The datasets presented in this study can be found in online repositories. The names of the repository/repositories and accession number(s) can be found in the article/**Supplementary Material**.

## Author contributions

ZX, TZ, JiaL, CW, and HH conceived the project. ZX, TZ, JinjL, and HH conducted the experiments. ZX, TZ, JiaL, LZ, JinjL, RZ, JLU, YW, CW, and HH discussed the experiment. ZX, TZ, JiaL, and HH analyzed and interpreted the data. ZX and HH prepared the figures. ZX and HH prepared the manuscript draft. ZX and TZ wrote the article with input from all authors. All authors contributed to the article and approved the submitted version.
