# Supplementary material for: TcbZIP60 positively regulates pyrethrins biosynthesis in Tanacetum cinerariifolium
Source: Front Plant Sci. 2023 Feb 20;14:1133912. doi: 10.3389/fpls.2023.1133912 (PMC9986458; doi:10.3389/fpls.2023.1133912)
Supplement: Supplementary file 2 [file DataSheet_2.docx]

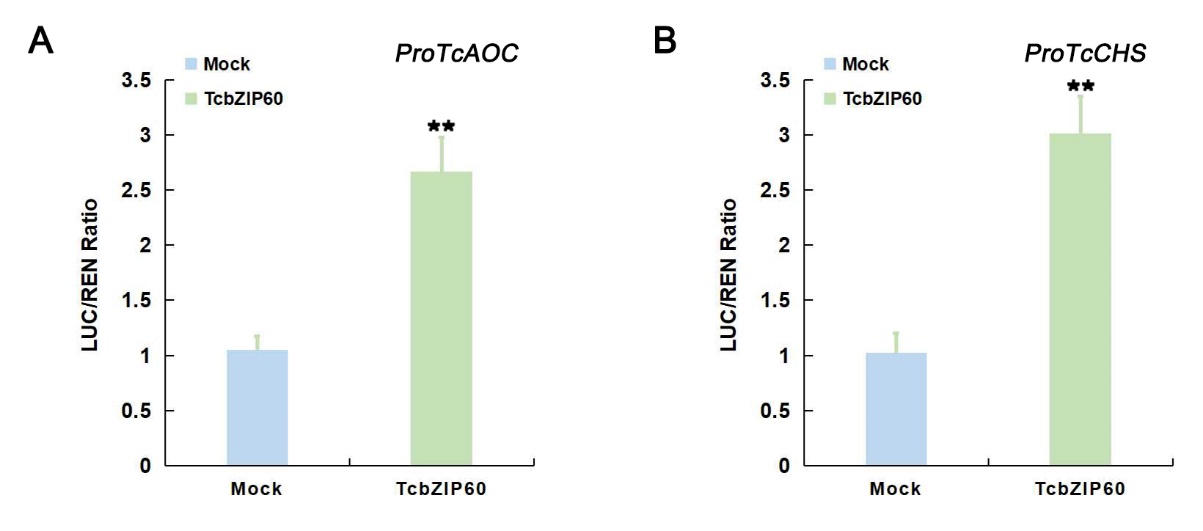


**Figure S1. Dual-LUC transient expression assay**. Dual-LUC transient expression assay showing the *TcCHS*/*TcAOC* promoters activity in tobacco, based on the LUC/REN ratios. LUC/REN of the control, in the absence of the effector, was considered as 1. Asterisks indicate that the value is significantly different from that of the control (**P<0.01).


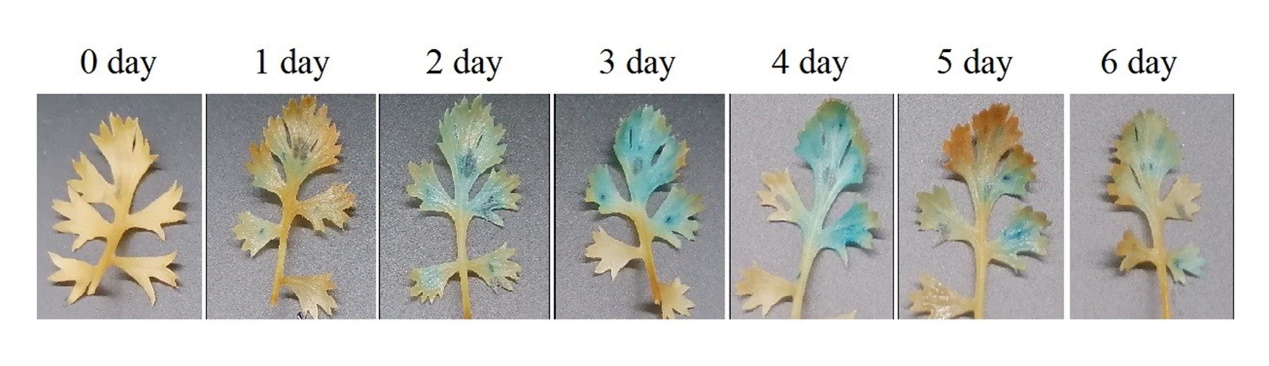


**Figure S2. Transient overexpression of GUS gene in leaves**. After staining with X-Gluc reagent, it was found that *T. cinerariifolium* leaves with transient overexpression of GUS gene had the best staining effect on days 3 and 4, indicating that the expression of foreign genes peaked on days 3 and 4.

**Table S1. Primers used in experiments.**

| ID | Primer name | Primer sequences（5' to 3'） |  |
| --- | --- | --- | --- |
| Cloning | TcbZIP60_ORF_F | ATGGAAGAAGAAAATATGATGATCG |  |
| Cloning | TcbZIP60_ORF_R | CTAATAAGAAGTTACTACTTTTAGCG |  |
| Realtime PCR | TcCHS_RT_F | ACGTGCATCTTCTGGACCTCTTC |  |
| Realtime PCR | TcCHS_RT_R | TGAACAATCCGACGGTTAAGAGTC |  |
| Realtime PCR | TcGLIP_RT_F | GCCGGGAATGCGAGCAAAACAAC |  |
| Realtime PCR | TcGLIP_RT_R | CGCTCTCGCCTTCCTTAAAACCATA |  |
| Realtime PCR | TcAOC_RT_F | ATCGTGGAAGTCCTGCTTATCTAC |  |
| Realtime PCR | TcAOC_RT_R | AATGGCTTCGTATCTGTCACCTT |  |
| Realtime PCR | TcALDH_RT_F | CATTCCGCTACTTTGCTGGTGC |  |
| Realtime PCR | TcALDH_RT_R | TCCAAGGAATGATGTGTCCAACTAC |  |
| Realtime PCR | TcGAPDH_RT_F | AAGGAGGAATCTGAAGGAAAGCTG |  |
| Realtime PCR | TcGAPDH_RT_R | GTTGTTGTTCAAAGCGATTCCAGC |  |
| Realtime PCR | TcbZIP60_RT_F | TCTACTTCTCCTGATGTTGTTG |  |
| Realtime PCR | TcbZIP60_RT_R | TTCCTCTTCATAGTACCTGCTT |  |
| linking to pHis2.1 vector | TcCHS_Pro_pHis_F | GACTCACTATAGGGCGAATTCGCTATTATAAAATCCCGTGTCTATGC |  |
| linking to pHis2.1 vector | TcCHS_Pro_pHis_R | ATTACTAGTGGATCCACGCGTCATTTACAACAGAATCTTAATGTGAGTGT |  |
| linking to pHis2.1 vector | TcAOC_Pro_pHis_F | GACTCACTATAGGGCGAATTCTATTTAACTTGTATATATACATGGGTTGAAGC |  |
| linking to pHis2.1 vector | TcAOC_Pro_pHis_R | ATTACTAGTGGATCCACGCGTAGTTGTTAAGATTTGTTTTAATGTTTAATGC |  |
| linking to pGADT7 vector | bZIP60_pGADT7_F | GTACCAGATTACGCTCATATGATGGAAGAAGAAAATATGATGATCGA |  |
| linking to pGADT7 vector | bZIP60_pGADT7_R | ACGATTCATCTGCAGCTCGAGCTAATAAGAAGTTACTACTTTTAGCGGAGA |  |
| linking to pSuper1300GFP vector | bZIP60_s1300g_F | GGGCCCGGGGTCGACATTTAAATATGGAAGAAGAAAATATGATGATCGA |  |
| linking to pSuper1300GFP vector | bZIP60_s1300g_R | GCCCTTGCTCACCATGGTACCATAAGAAGTTACTACTTTTAGCGGAGAATC |  |
| linking to pGreenⅡSK62 vector | bZIP60_SK62_F | CAGGAATTCGATATCAAGCTTATGGAAGAAGAAAATATGATGATCGA |  |
| linking to pGreenⅡSK62 vector | bZIP60_SK62_R | GTCGACGGTATCGATAAGCTTCTAATAAGAAGTTACTACTTTTAGCGGAGA |  |
| linking to pGreenⅡ0800 LUC vector | CHS_LUC_F | GTCGACGGTATCGATAAGCTTGCTATTATAAAATCCCGTGTCTATGC |  |
| linking to pGreenⅡ0800 LUC vector | CHS_LUC_R | CAGGAATTCGATATCAAGCTTTTACAACAGAATCTTAATGTGAGTGTATGT |  |
| linking to pGreenⅡ0800 LUC vector | AOC_LUC_F | GTCGACGGTATCGATAAGCTTTATTTAACTTGTATATATACATGGGTTGAAGC |  |
| linking to pGreenⅡ0800 LUC vector | AOC_LUC_R | CAGGAATTCGATATCAAGCTTAGTTGTTAAGATTTGTTTTAATGTTTAATGC |  |
| linking to pET6HN-C vector | TcbZIP60_pET6N_F | GATCTCTAAGCTTGCGAATTCTATGGAAGAAGAAAATATGATGATCG |  |
| linking to pET6HN-C vector | TcbZIP60_pET6N_R | ACCAGGCGGCCGCCAGAATTCGCATAAGAAGTTACTACTTTTAGCG |  |
| Probes used in EMSA | Probe_proCHS _F | TTTGAAGGCAAGTGATGTAAAGTGCTAAGTGTTAAGTCAATGATTATAT |  |
| Probes used in EMSA | Probe_proCHS _R | ATATAATCATTGACTTAACACTTAGCACTTTACATCACTTGCCTTCAAA |  |
| Probes used in EMSA | Probe_proAOC_F | CAACTAATCACACACCGCCACGTGTCCACCGTCCACCTACT |  |
| Probes used in EMSA | Probe_proAOC_R | AGTAGGTGGACGGTGGACACGTGGCGGTGTGTGATTAGTTG |  |
| Probes used in EMSA | mProbe_proCHS _F | TTAAAAAGCAAAAAATGTAAAGTGCTAAGTGTTAAAAAAAAAATTATAT |  |
| Probes used in EMSA | mProbe_proCHS _R | ATATAATTTTTTTTTTAACACTTAGCACTTTACATTTTTTGCTTTTTAA |  |
| Probes used in EMSA | mProbe_proAOC_F | CAACTAATCACACACCGCTCTTCTTCCACCGTCCACCTACT |  |
| Probes used in EMSA | mProbe_proAOC_R | AGTAGGTGGACGGTGGAAGAAGAGCGGTGTGTGATTAGTTG |  |
| ptrv1_check_F | PTRV1_3807_F | GGCCTTGCGCCGTTCCAGAT |  |
| ptrv1_check_R | PTRV1_4667_R | CCCAAAGGAAGGCCGCCCAC |  |
| ptrv2_check_F | PTRV2_1604_F | TTATTACGGACGAGTGGACTTAG |  |
| ptrv2_check_R | PTRV2_1761_R | AACTTCAGACACGGATCTACTT |  |
| linking to pTRV2 vector | TcbZIP60_VIGS_F | AGAAGGCCTCCATGGGGATCCGATCCGTGCTCCAATGCTTT |  |
| linking to pTRV2 vector | TcbZIP60_VIGS_R | CGTGAGCTCGGTACCGGATCCTGAATCGTTTACCCACCATAAGG |  |

**Table S2. Part of cis-acting regulatory elements in promoters of *TcCHS, TcAOC,TcALDH, TcGLIP.***

| Gene Name | Motif-Name | Core sequence | Num | Predictive function |
| --- | --- | --- | --- | --- |
| TcALDH  TcAOC  TcCHS  TcGLIP | ABRE3a  ABRE  ABRE4  as-1  CGTCA-motif  G-box  ABRE3a  TGACG-motif  G-box  as-1  GARE-motif  E-box  TC-rich repeats  as-1  TC-rich repeats  ABRE4 | TACGTG  GCAACGTGTC  CACGTA  TGACG  CGTCA  CACGAC  TACGTG  TGACG  ACACGTGGC  TGAC  TCTGTTG  CAAGTG  GTTTTCTTAC  TGAC  ATTCTCTAAC  CACGTA | 6  9  6  5  5  6  6  5  8  5  7  5  9  9  9  6 | cis-acting element involved in the ABA responsiveness  cis-acting element involved in the ABA responsiveness  cis-acting element involved in the ABA responsiveness  cis-acting element involved in the SA responsiveness  cis-acting element involved in the MeJA responsiveness  cis-acting regulatory element involved in light responsiveness  cis-acting element involved in the ABA responsiveness  cis-acting element involved in the MeJA responsiveness  cis-acting regulatory element involved in light responsiveness  cis-acting element involved in the SA responsiveness  gibberellin-responsive element  binding site of bZIP binding protein  cis-acting element involved in defense and stress responsiveness  cis-acting element involved in the SA responsiveness  cis-acting element involved in defense and stress responsiveness  cis-acting element involved in the ABA responsiveness |
|  | G-box | TACGTG | 6 | cis-acting regulatory element involved in light responsiveness |

**Table S3.** **Genbank accession of the genes used in the assay**

| Name | GenBank accession | Species |
| --- | --- | --- |
| AtbZIP60 | AT5G11260.1 | *Arabidopsis thaliana* |
| AaHY5 | PWA35981.1 | *Artemisia annua* |
| TcbZIP60 | OM988163 | *Tanacetum cinerariifolium* |
| AtHY5 | AT5G11260 | *Citrus unshiu* |
| NbbZIP60 | QBA30963.1 | *Nicotiana benthamiana* |
| NabZIP60-like | XP_019252016.1 | *Nicotiana attenuata* |
| ZmbZIP60 | PWZ40661.1 | *Zea mays* |
| OsbZIP60 | XP_015647746.1 | *Oryza sativa* |
| GmbZIP60 | ABI34648.1 | *Glycine max* |
| JcbZIP60-like | XP_022867730.1 | *Jatropha curcas* |
